# Differential Impact of Visuospatial Working Memory on Rule-based and Information-integration Category Learning

**DOI:** 10.3389/fpsyg.2017.00530

**Published:** 2017-04-07

**Authors:** Qiang Xing, Hailong Sun

**Affiliations:** ^1^Department of Psychology, Guangzhou UniversityGuangzhou, China; ^2^Management School, Jinan UniversityGuangzhou, China

**Keywords:** visuospatial working memory, visual processing, rule-based category structure, information-integration category structure, executive function, dual-task paradigm

## Abstract

Previous studies have indicated that the category learning system is a mechanism with multiple processing systems, and that working memory has different effects on category learning. But how does visuospatial working memory affect perceptual category learning? As there is no definite answer to this question, we conducted three experiments. In Experiment 1, the dual-task paradigm with sequential presentation was adopted to investigate the influence of visuospatial working memory on rule-based and information-integration category learning. The results showed that visuospatial working memory interferes with rule-based but not information-integration category learning. In Experiment 2, the dual-task paradigm with simultaneous presentation was used, in which the categorization task was integrated into the visuospatial working memory task. The results indicated that visuospatial working memory affects information-integration category learning but not rule-based category learning. In Experiment 3, the dual-task paradigm with simultaneous presentation was employed, in which visuospatial working memory was integrated into the category learning task. The results revealed that visuospatial working memory interferes with both rule-based and information-integration category learning. Through these three experiments, we found that, regarding the rule-based category learning, working memory load is the main mechanism by which visuospatial working memory influences the discovery of the category rules. In addition, regarding the information-integration category learning, visual resources mainly operates on the category representation.

## Introduction

Categorization is a fundamental decision-making process that allows us to meaningfully parse the world and group similar objects together so that they can be treated equivalently (Rabi and Minda, 2014). It enables us to apply what we have learned about one thing and generalize that knowledge to other things of the same kind. For example, after learning the hard way that a particular mushroom is probably poisonous, it is highly adaptive to generalize that knowledge to other similar mushrooms rather than to have to learn the hard way every time a new mushroom is encountered (Richler and Palmeri, 2014).

A large number of studies have indicated that category learning contains multiple classes of processing systems (Maddox et al., 2004; Richler and Palmeri, 2014; Xing and Sun, 2015), which have been explained by different theoretical models, such as exemplar-similarity (Patalano et al., 2001), family-resemblance (Yamauchi and Markman, 1998), and so on. The COVIS is so far the most influential multi-system theory, according to which there are at least two independent systems that exist in human category learning. One is the verbal system that is based on hypothesis testing and is under the control of consciousness, which is also influenced by working memory. The other is an implicit system that solves categorization tasks by learning to associate a response with regions of perceptual space, which is based on reinforcement and is independent of working memory (Ashby et al., 1998).

**FIGURE 1:**
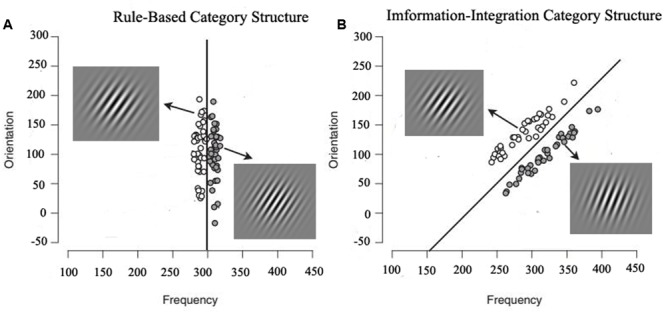
**The RB category structure and II category structure.** Open circles denote Category **(A)** and filled circles denote Category **(B)**. The lines represent the optimal decision boundary. In a RB category structure, decisions are made based on only one dimension (in this example, frequency), whereas in an II category structure, decisions are made based on two or more dimensions (in this example, frequency and orientation).

This has led to an extensive series of studies that have compared the learning of RB and II category structures (**Figure 1**). Given that the categorization of the RB and II structures depends primarily on the verbal and implicit systems, respectively, it is possible to test two kinds of prediction made by the COVIS model (Dunn et al., 2012). For the RB category structure, the classification rules are easy to verbalize and a judgment rule does not require the integration of two dimensions. For example, consider a category set in which round objects belong to one group and square objects belong to another group. These categories could be learned by applying the easy to verbalize rule that “category 1 objects are round.” However, in contrast, the II category structure defines category membership according to the conjoint values on two or more dimensions using rules that are not easy to verbalize (e.g., if the size of a circle is greater than *x* and the orientation of a line is greater than *y*, then the stimulus is a member of category A). Consequently, such structures cannot be learned by the verbal system, which must eventually yield control of the response to the implicit system (Maddox et al., 2004; Worthy et al., 2013; Richler and Palmeri, 2014).

Furthermore, according to the COVIS model, working memory involves the ability to store information transiently and to perform cognitive activities, and it has different effects on the RB and II category structures. In other words, if working memory tasks are presented concurrently, the RB category learning will be disturbed, while the II category learning will not be affected; this has been verified by a large number of studies (Maddox et al., 2004; Zeithamova and Maddox, 2006; Filoteo et al., 2010). It is worth noting that the experiments mentioned above all involved verbal working memory. However, visuospatial working memory is another important type of working memory (Baddeley and Logie, 1999). There is good evidence that verbal and visuospatial working memory rely on different neural systems (Goldman-Rakic, 1998). Compared with verbal working memory, visuospatial working memory not only includes working memory load but also involves visual resources. Additionally, processing in the implicit system depends critically on the visual stimulus’s representation in the inferotemporal cortex. This representation may be disrupted by the presence of a visuospatial task (Casale and Ashby, 2008). Thus, one hypothesis is that the presence of a visuospatial working memory task will affect II category learning. However, the existing COVIS model does not distinguish the types of working memory, and previous studies have mainly focused on the effects of verbal working memory on RB and II category learning. As such, the question naturally arises of how visuospatial working memory affects the RB and II category learning.

Using the dual-task paradigm that involves simultaneous presentation of visual stimuli, Miles and Minda (2011) found that a high level of working memory load could impair the RB category learning, which confirms that working memory plays a significant role in the RB category learning. In addition, their study found that the visual processing of the visuospatial working memory task affected the II category learning, which was not related to the level of working memory load. However, the study used the RB category structure that relies on a single dimension to perform categorization, whereas the II category structure takes two dimensions into consideration; thus, the difference in the difficulty of the category structure could affect the categorization results (Sun and Xing, 2014; Zaki and Kleinschmidt, 2014). Miles and Minda (2011) believe that visuospatial working memory mainly depends on a function that influences implicit category learning, which is not related to working memory load. Nevertheless, the study did not explain how visual processing affects category learning. Moreover, Miles et al. (2014) used a simultaneous-task paradigm in which the category learning task is integrated with a verbal working memory task, and the results showed that working memory load can affect II category learning.

It can be seen that there is still much debate about the influence of visuospatial working memory on the RB and II category structure, especially about how visuospatial working memory affects II category learning. If it does affect II structure category learning, would there be any difference in the results of the above-mentioned studies? Several studies may offer some insight to solve these problems. According to the hypothesis of Zeithamova and Maddox (2007), the process of category learning may include the following steps: (1) representation of the stimulus and (2) generation and testing of a categorization rule for the RB category learning (i.e., learning of a categorization criterion for the II category learning). Thus, we suppose that if the process of category learning really includes these steps, the working memory load from the visuospatial working memory task would be critical primarily for rule generation and testing (because the verbal system depends upon working memory load), while the visuospatial resource from the visuospatial working memory task may influence the representation of the stimulus for the II category learning (because the implicit system learns the association between a region of perceptual space and an overt response).

In practical terms, the implicit category learning system establishes a connection between a specific perceptual space and the specific action, and the representation of the category stimuli is involved in the category learning. This is indicated in the study by Dunn et al. (2012) in which a Gabor mask presented after the II category structure interfered with the visual processing of the category stimuli and affected the perceptual representation of the II category learning. Maddox et al. (2004) found that the addition of a working memory load in the sequential presentation impaired RB learning but had little effect on II learning. Furthermore, when studying the effects of working memory on category learning, dual-task paradigms are usually adopted, such as the dual task with sequential presentation and the dual task with simultaneous presentation, in which the different locations of working memory are manipulated (Miles and Minda, 2011).

Therefore, the inconsistencies in the previous studies are much more likely to be caused by the fact that visual resources and working memory load may affect the different processing stages of category learning. Based on this point of view, we conducted three experiments in which we manipulated the different dual tasks in order to examine whether they would influence the different cognitive processing stages of category learning. We aimed to investigate the process of cognitive processing during which visuospatial working memory affects II and RB category learning, especially visual resources and working memory. In Experiment 1, sequential presentation tasks were adopted. In Experiment 2, we used the embedded paradigm in which the category learning task was embedded in the visuospatial working memory task. In Experiment 3, we used a concurrent-task methodology in which the working memory task was embedded in the classification task.

## Experiment 1

### Materials and Methods

#### Participants

We randomly selected 84 participants (40 male, 44 female) who were participating in the secondary post-graduate examination held by the education school of Guangzhou University. The average age was 19.31 years (±2.15). All participants were right-handed, had normal or corrected-to-normal vision, and had no color blindness or color weakness problems. This study was carried out in accordance with the recommendations of the ethical committee of Guangzhou University with written informed consent from all participants. All participants gave written informed consent in accordance with the Declaration of Helsinki.

#### Experimental Materials

The categorization stimuli were generated using the same procedures as Dunn et al. (2012). The stimuli were sine wave gratings that varied in spatial frequency and orientation. Twenty stimuli in each of the four categories were generated by sampling randomly from the same four parameter distributions used by Dunn et al. (2012). The Psychophysics Toolbox in MATLAB (MathWorks, Natick, MA, USA) was used to generate the RB and II category structures (Brainard, 1997). Actual values of spatial frequency (*f*) and orientation (*o*) were generated from a random sample (*x, y*) from these distributions using the following transformations: *f* = 0.25 + *x*/50, *o* = *y.*π/500. All of the stimuli were 200 × 200 pixel images. The specific dimensions of the parameters are shown in **Table 1**.

**Table 1:** Rule-based and II category structure parameters.

Category structure	μ_X_	μ_Y_	σ^2^_X_	σ^2^_Y_	*Cov*
**RB**					
Category A	268	93	75	75	0
Category B	268	157	75	75	0
Category C	332	93	75	75	0
Category D	332	157	75	75	0
**II**					
Category A	268	125	75	75	0
Category B	300	157	75	75	0
Category C	300	93	75	75	0
Category D	332	152	75	75	0

*μ_*X*_ represents the average value, σ^*2*^_*X*_ represents the variance, cov represents the variance covariance, RB represents the rule-based category structure, and II represents the information-integration category structure. According to the frequency and direction, each category structure was divided into four categories: A, B, C, and D.*

A visuospatial working memory task was created that was analogous to the Sternberg working memory task used in Maddox et al. (2004). In this task, the participant was asked to remember four locations out of nine possible locations (analogous to remembering four numerical digits sampled from nine possible digits). First, a fixation cross (i.e., a “+”) appeared in the middle of the screen, indicating the beginning of the dot pattern task. Next, nine gray dots appeared on the screen and the memory set turned red, followed by a series of four rapidly presented masks. Each mask was a 9 × 9 grid of gray dots, half of which had a red center. Next, the memory probe appeared on the screen along with the question “Was this dot originally red?” Participants made a response using the appropriate button and received feedback.

#### Experimental Design

The experiment had a 2 (task condition: working memory group vs. control group) × 2 (category structure: II vs. RB) × 4 (block) repeated-measures design, in which task condition and category structure were the between-subjects variables and learning block was the within-subjects variable. The dependent variables were the accuracy of categorization in the visuospatial working memory task and the category learning. The number of participants followed that used by previous studies (Stanton and Nosofsky, 2007; Miles and Minda, 2011). All participants were assigned randomly to one of four groups, with 21 participants in each group. Two participants in the RB task control group (RB-C) were removed due to interruption during the experiment; thus, the data from 19 participants were used. One participant in the RB task experimental group (RB-V) was removed for the same reason; therefore, the data of 20 participants were used. The data of 21 participants were used in the II task experimental group (II-V), while that of 19 participants were used in the II control group (II-C) after deleting the data of two participants for the same reason.

#### Experimental Procedure

**FIGURE 2:**
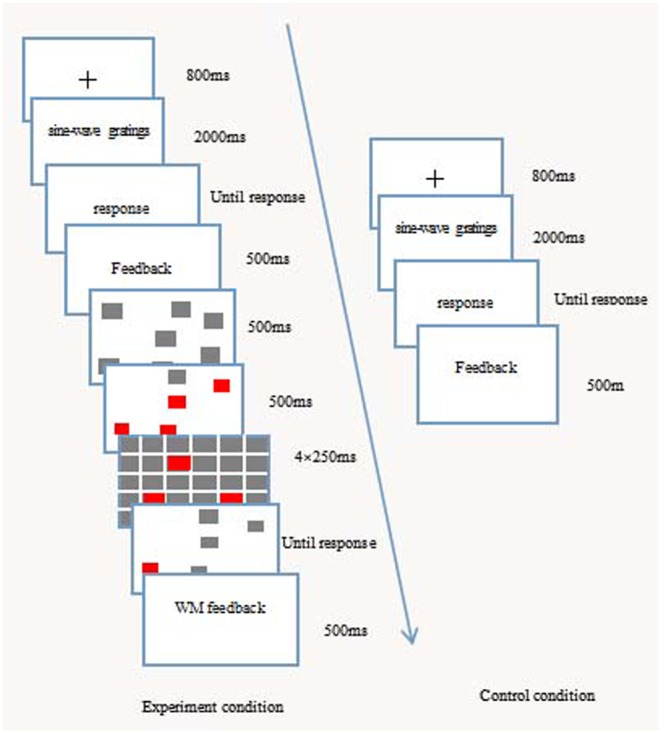
**The experimental flow chart of the category learning and visuospatial working memory tasks, under the condition of sequential presentation**.

The dual-task experimental paradigm with sequential presentation was used (**Figure 2**). The experimental procedure included four blocks, each of which had 80 trials. First, participants tried to complete the category learning task. Within each block, all 80 stimuli were presented in a random order. Participants were told to learn which of four categories (labeled as 1, 2, 3, and 4) each stimulus belonged to. After the presentation of a fixation cross (i.e., a “+”) for 800 ms, the screen was presented of the RB or II category structure, which could be considered by participants as belonging to one of the four categories of A, B, C, or D, and for which they pressed the 1, 2, 3, or 4 number key on the keyboard, respectively. After the responses were given, the stimuli disappeared, and the feedback was provided immediately; the participants were informed not only whether their responses were correct or not, but also to which category each of the stimuli belonged, and the correct sine wave grating was shown to the participants at the same time.

The visuospatial working memory task followed the category learning task. The gray squares were presented on the screen for 500 ms. Then, four randomly selected gray squares all turned red for 500 ms before disappearing. After this, another gray square turned red (which could be one of the four squares that had changed color from gray to red or it could be a new square), followed by a series of four quickly presented masks. Each mask was a 9 × 9 grid of gray squares, half of which had a red center. The participants were required to determine whether this square had appeared before or not. If they believed that it had been presented before, they pressed the “F” key. If they believed that it had not been presented before, they pressed the “J” key. After the responses were given, the participants were provided with feedback for 800 ms about whether they were right or wrong. In contrast, the control group was not presented with the visuospatial working memory task. They were required to only perform the category learning task, which was the same as for the experimental group.

### Results

#### Visuospatial Working Memory Task Performance

The mean accuracy rates averaged across participants were analyzed. The average accuracy of the visuospatial working memory task in the RB-V group was 0.71 (±0.18), and that of the participants in the II-V group was 0.71 (±0.17). An independent-samples *t*-test showed that there was no significant difference between the two groups, *t*(38) = -0.097, *p* = 0.977, indicating that there was no difference in the degree of cognitive resources consumed by participants in the RB group and II group when performing the visuospatial working memory task.

#### Analysis of the Overall Results

**FIGURE 3:**
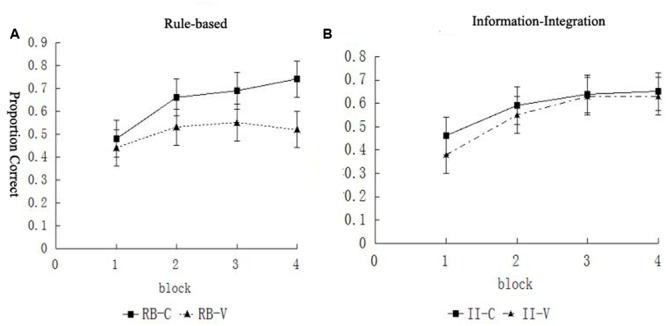
**The categorization accuracy of the (A)** RB and **(B)** II category structures in the different blocks.

We conducted a 2 (category structure) × 2 (condition) × 4 (block) mixed design analysis of variance. This revealed a main effect of block, *F*(3,225) = 57.28, *p* < 0.001, $\eta_{p}^{2}$= 0.43, indicating learning, and a main effect of condition, *F*(1,75) = 4.24, *p =* 0.043, $\eta_{p}^{2}$ = 0.05, indicating superior accuracy overall for the control condition compared to the visuospatial working memory condition. There was no main effect of category structure, *F <* 1, and no significant interactions between block and category structure, *F*(3,225) = 1.37, *p =* 0.253, between block and condition, *F*(3,225) = 1.27, *p =* 0.287, or between category structure and condition, *F*(1,94) = 1.42, *p =* 0.237. However, there were significant interactions between block, category structure, and condition, *F*(3,225) = 4.53, *p =* 0.004, $\eta_{p}^{2}$ = 0.06. The interactions with category structure indicate that the condition had a greater effect on RB learning than II learning and that this difference increased across the blocks (**Figure 3**).

Furthermore, for the RB category structure, a 2 (condition) × 4 (block) repeated-measures analysis of variance was performed (**Table 2**). The results showed that the main effect of the block was significant, *F*(3,111) = 18.45, *p* < 0.001, $\eta_{p}^{2}$ = 0.33, indicating that learning occurred. The significant main effect of the condition, *F*(1,37) = 4.45, *p* = 0.042, $\eta_{p}^{2}$ = 0.11, indicated that the participants’ learning was significantly different in the different conditions. Furthermore, the interaction between the condition and block was significant, *F*(3,111) = 4.50, *p* = 0.005, $\eta_{p}^{2}$ = 0.11, indicating that, in the two conditions, the findings of the different blocks were significantly different. The analysis of the simple effects showed that the difference in results between the experimental group and control group was not significant in Block 1 (*p* = 0.553). In Block 2, the results of the RB task categorization of the experimental group were significantly lower than those of the control group (*p* = 0.050). In Block 3, the results of the categorization task of the experimental group were not significantly different from those of the control group (*p =* 0.077). In Block 4, the results of the categorization task of the experimental group were significantly lower than those of the control group (*p* = 0.008) (**Figure 3**). All of these findings indicate that performing the visuospatial working memory task immediately after the feedback impaired the RB category learning.

**Table 2:** Effects of working memory on the II and RB category structures (*M ± SD*).

	1	2	3	4
RB-C	0.48 ± 0.18	0.66 ± 0.22	0.69 ± 0.25	0.74 ± 0.25
RB-V	0.44 ± 0.16	0.53 ± 0.20	0.55 ± 0.23	0.52 ± 0.24
II-C	0.46 ± 0.14	0.59 ± 0.18	0.64 ± 0.22	0.65 ± 0.20
II-V	0.38 ± 0.18	0.55 ± 0.17	0.63 ± 0.18	0.63 ± 0.20

*RB and II represent the ule-based category structure and the information-integration category structure, respectively. RB-C represents the rule-based category structure control group, RB-V represents the rule-based category structure experimental group, II-C represents the information-integration Category structure control group, and II-V represents the information-integration category structure experimental group. The numbers 1, 2, 3, and 4 represent the four blocks of the learning process.*

For the II category structure, the 2 (condition) × 4 (block) repeated-measures analysis of variance was performed in the same way. The results showed that the main effect of the block was significant, *F*(3,114) = 43.53, *p* < 0.001, $\eta_{p}^{2}$ = 0.53, indicating that learning occurred. The main effect of the condition was not significant, *F*(1,38) = 0.46, *p* = 0.503, and the interaction between the condition and block was not significant, *F*(3,111) = 0.75, *p* = 0.525 (**Figure 3**). All of these findings indicate that the visuospatial working memory task conducted immediately after the feedback did not influence the II category learning.

To summarize, we found that a visuospatial working memory task interferes with RB but not II category learning when the dual-task experimental paradigm with sequential presentation is used. The significant effect of visuospatial working memory on RB category learning replicates the effect observed in Maddox et al. (2004) with a verbal working memory task, and extends the effect to a visuospatial working memory task. As outlined in the introduction, the RB learning involves generating a representation of the stimulus, response, and feedback. Thus, placing a load on a separate visuospatial working memory store will affect the feedback processes. In contrast, the II category learning appears to occur incrementally in a fashion that is heavily dependent on immediate feedback. As such, would a nested form of visual working memory affect category learning? In Experiment 2, we used an embedded paradigm in which the category learning task was embedded in the visuospatial working memory task in order to examine the effect of the working memory on RB and II category learning.

## Experiment 2

### Materials and Methods

#### Participants

We randomly selected 87 students (40 male, 47 female) who were participating in the secondary post-graduate examination held by the education school of Guangzhou University. The average age was 19.61 years (±1.62). Twenty participants were assigned to the RB-C condition, 24 to the RB-V condition, and 22 and 21 to the II-V condition and the II-C condition, respectively. We aimed for 20 participants per condition based on previous studies, such as Miles and Minda (2011). All of the participants were right-handed, had normal or corrected-to-normal vision, and had no color blindness or color weakness problems. Written informed consent was obtained from all participants before starting the investigation in accordance with the Declaration of Helsinki, and the study was approved by the ethical committee of Guangzhou University.

#### Experimental Materials

These were the same as in Experiment 1.

#### Experimental Design

This was the same as in Experiment 1.

#### Experimental Procedure

**FIGURE 4:**
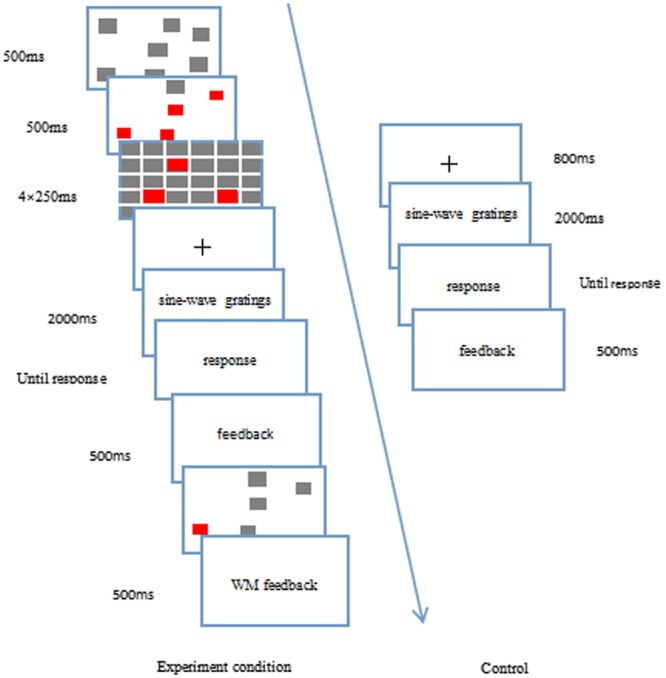
**The experimental flow chart of the category learning task when it was embedded in the visuospatial working memory task**.

The dual-task experimental paradigm with simultaneous presentation was employed in which the category learning task was integrated into the visuospatial working memory task (**Figure 4**). The whole experimental process was divided into three stages.

In the first stage, the gray squares were presented for 500 ms. Four random squares of the screen then turned red for 500 ms, after which the screen disappeared. The masking appeared four times in sequence, each one lasting for 250 ms (1000 ms in total), in which random flickering squares were presented on each screen. The participants did not have to respond during this stage.

The second stage was the category learning task. A fixation cross (i.e., a “+”) was presented for 800 ms, after which it disappeared. The screen then showed the RB or II category structure for 200 ms, after which it disappeared, and the response screen of the category learning was presented, in which there were four categories (i.e., A, B, C, and D). The participants determined which category structure it belonged to and pressed the relevant key on the keyboard for each category (i.e., 1, 2, 3, and 4, respectively). The screen disappeared after the responses were given, and the instant feedback was then provided.

In the third stage, after finishing the category learning task, the gray squares were randomly presented on the screen for 500 ms, followed by the detection screen, in which a gray square turned red (which may have occurred in the first stage or not) and the participants were required to determine whether this square had appeared before or not. If the participant believed that it had been presented before, they pressed the “F” key. If the participant believed that it had not been presented before, they pressed the “J” key. The detection screen disappeared after the responses were given, and the simple feedback was then provided. The whole experimental procedure included four blocks, each of which had 80 trials. If the participant’s performance on the visuospatial working memory task in each block was lower than 80%, a warning window popped up at the end of the block. For the control group, there was no visuospatial working memory task and participants were required to only perform the category learning task, which was the same as for the experimental group.

### Results

#### Concurrent Task Performance

The average accuracy for the visuospatial working memory task of participants in the RB-V group was 0.72 (±0.15), and that of participants in the II-V group was 0.68 (±0.11). An independent-samples *t*-test showed that there was no significant difference between the two groups, *t*(44) = -0.943, *p =* 0.320, indicating that there was not a difference in the degree of cognitive resources consumed by participants in the RB and II groups when performing the visuospatial working memory task.

#### Analysis of the Category Learning

**FIGURE 5:**
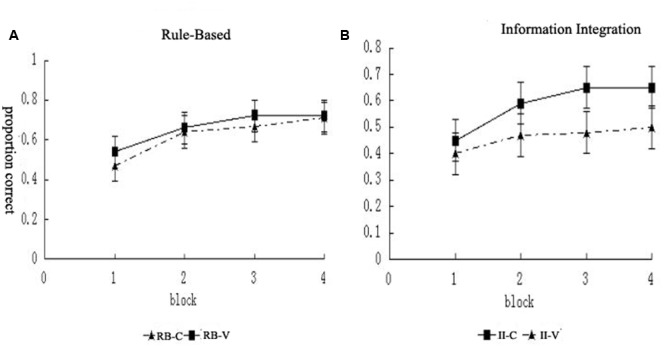
**The learning curves of participants during the different blocks in the (A)** RB and **(B)** II conditions.

We conducted a 2 (category structure) × 2 (condition) × 4 (block) mixed design analysis of variance. This revealed a main effect of block, *F*(3,249) = 62.33, *p* < 0.001, $\eta_{p}^{2}$ = 0.43, indicating learning, and a main effect of category structure, *F*(1,83) = 8.98, *p =* 0.004, $\eta_{p}^{2}$ = 0.10, indicating superior accuracy overall for the RB category structure compared to the II category structure. There was no main effect of condition, *F*(1,83) = 1.20, *p* = 0.276, and no significant interactions between block and category structure, *F*(3,249) = 2.26, *p =* 0.082, or between block, category structure, and condition, *F <* 1. However, there were significant interactions between block and condition, *F*(3,249) = 2.90, *p =* 0.036, $\eta_{p}^{2}$ = 0.03 and between category structure and condition, *F*(1,83) = 3.99, *p =* 0.049 (**Figure 5**).

Furthermore, for the RB category structure, we performed a 2 (condition) × 4 (block) repeated-measures analysis of variance (**Table 3**). The results showed that the main effect of the block was significant, *F*(3,126) = 34.36, *p* < 0.001, $\eta_{p}^{2}$ = 0.45, indicating the existence of a learning effect. The main effect of the condition was not significant, *F*(1,42) = 0.33, *p =* 0.567. In addition, the interaction between the condition and block was not significant, *F*(3,126) = 0.81, *p =* 0.493. All of these findings indicate that the visuospatial working memory task did not affect the RB category learning.

**Table 3:** Effects of visuospatial working memory on the II and RB category structures under the condition of simultaneous presentation (*M ± SD*).

	1	2	3	4
RB-C	0.47 ± 0.18	0.64 ± 0.22	0.67 ± 0.26	0.71 ± 0.27
RB-V	0.54 ± 0.16	0.66 ± 0.23	0.72 ± 0.23	0.72 ± 0.23
II-C	0.45 ± 0.14	0.59 ± 0.18	0.65 ± 0.21	0.65 ± 0.20
II-V	0.40 ± 0.14	0.47 ± 0.17	0.48 ± 0.17	0.50 ± 0.20

For the II structure, the 2 (condition) × 4 (block) repeated-measures analysis of variance showed that the main effect of the block was significant, *F*(3,123) = 29.07, *p* < 0.001, $\eta_{p}^{2}$ = 0.42, indicating the existence of a learning effect. The main effect of the condition was significant, *F*(1,41) = 6.17, *p* = 0.017, $\eta_{p}^{2}$ = 0.11, as was the interaction between the condition and block, *F*(3,123) = 4.03, *p* = 0.009, $\eta_{p}^{2}$ = 0.09, indicating that in these two conditions, the results of the different blocks were significantly different.

In order to further investigate the interaction between the conditions and blocks in detail, an analysis of the simple effects was conducted. The results showed that, in Block 1, there was no significant difference in the results between the II-C and II-V groups, *p* = 0.200; in Block 2, the results of the II-C group were significantly higher than those of the II-V group, *p* = 0.027; in Block 3, the results of the II-C group were significantly higher those of the II-V group, *p* = 0.008; and in Block 4, the results of the II-C group were significantly higher than those of the II-V group, *p* = 0.015 (**Figure 5**).

In Experiment 2, the dual-task paradigm with simultaneous presentation was used, in which the categorization task was integrated into the working memory task. The results indicated that visuospatial working memory affects the II category learning but not the RB category learning. On the contrary, the RB category learning was impaired by the visuospatial working memory task in Experiment 1. Due to there being a similar visuospatial working memory task, the two studies should have found the same effect of the visuospatial working memory task on RB and II category learning (according to the COVIS model, the study results are only affected by the degree of working memory load). Yet, the results of Study 2 showed different patterns of interference with the II category learning and RB category learning compared to the results of Study 1. These results are not explained by the COVIS model. However, Studies 1 and 2 differed in the location of the working memory task. We infer that the visuospatial resource may interfere with the perception of the stimuli. Experiment 2 provides the first piece of evidence that visuospatial working memory affects II category learning. This observed effect is consistent with Zeithamova and Maddox’s (2007) hypothesis of the stages of cognitive processing.

## Experiment 3

### Materials and Methods

#### Participants

We randomly selected 67 students (33 male, 34 female) who were participating in the secondary post-graduate examination held by the education school of Guangzhou University. The average age was 18.98 years (±0.79). There were 20 participants assigned to the RB-C condition, 23 to the RB-V condition, and 24 and 23 to the II-V condition and the II-C condition, respectively. All of the participants were right-handed, had normal or corrected-to-normal vision, and had no color blindness or color weakness problems. Written informed consent was obtained from all participants before starting the investigation in accordance with the Declaration of Helsinki, and the study was approved by the ethical committee of Guangzhou University.

#### Experimental Materials

These were the same as in Experiment 1.

#### Experimental Design

This was the same as in Experiment 1.

#### Experimental Procedure

**FIGURE 6:**
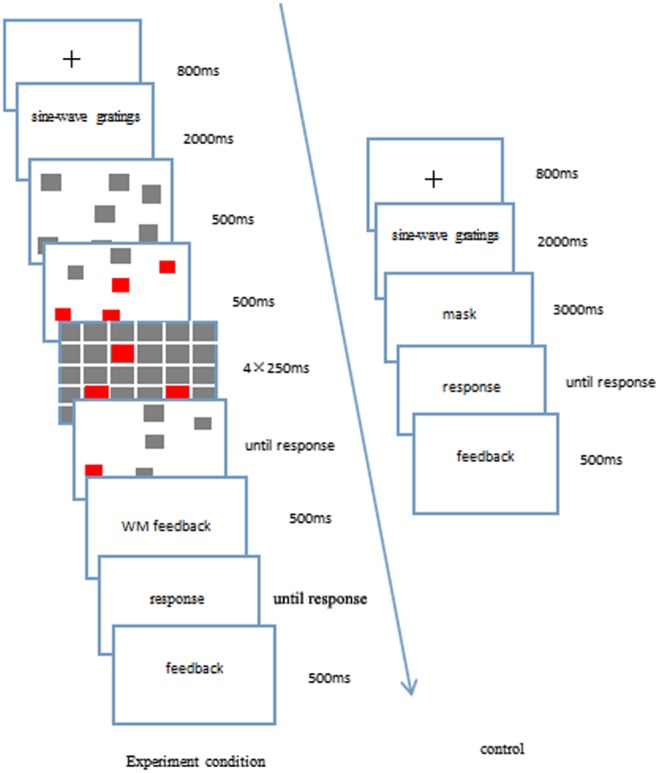
**The experimental flow chart of the working memory task when it was embedded in the category learning task**.

The dual-task experimental paradigm with simultaneous presentation was adopted for the visuospatial working memory experimental group, in which visuospatial working memory was integrated into the category learning. The whole experimental procedure was divided into three stages (**Figure 6**).

In the first stage, the fixation cross (i.e., a “+”) was presented for 800 ms, after which it disappeared. The screen then showed the RB or II category structure for 200 ms, after which it disappeared. During this stage, the participants were not required to respond.

The second stage included the visuospatial working memory task. The gray squares were randomly presented on the screen for 500 ms. Four random squares then changed color from gray to red, which lasted for 500 ms, after which the screen disappeared. The masking appeared four times in a row, with each lasting for 250 ms. After that, the detection screen was presented in which a gray square turned red (which may have appeared before or not) and the participants were required to determine whether this square had appeared before or not. If they believed that it had been presented before, the “F” key was pressed; otherwise, the “J” key was pressed. As soon as the responses were given, the detection screen disappeared, and the feedback about whether the participants were right or wrong was provided.

In the third stage, at the end of the visuospatial working memory task, the response screen of the category learning task was presented, in which there were four categories (i.e., A, B, C, and D). The participants decided to which category structure it belonged, and pressed the counterpart key on the keyboard (i.e., 1, 2, 3, and 4, respectively). The screen disappeared after the responses were given, and abundant feedback was provided instantly. The experimental procedure included four blocks, each of which had 80 trials. For the control group, there was no visuospatial working memory task, and participants were required to only perform the category learning task. There was a delay of 3000 ms (the shortest presentation time in the whole visuospatial working memory task) for the gray screen between the screen presenting the category structure and the response screen, so that it was the same as for the conditions of the experimental group. The category learning task of the control group was the same as that of the experimental group (**Figure 6**).

### Results

#### Concurrent Task Performance

The average accuracy of accomplishing the visuospatial working memory task of participants in the RB-V group was 0.84 (±0.14), and that of participants in the II-V group was 0.88 (±0.09). An independent-samples *t*-test showed that there was no significant difference between the two groups, *t*(45) = 1.16, *p* = 0.167, indicating that there was not a difference in the degree of cognitive resources consumed by participants in the RB and II groups when performing the visuospatial working memory task.

#### Analysis of the Overall Results for the Category Learning

**FIGURE 7:**
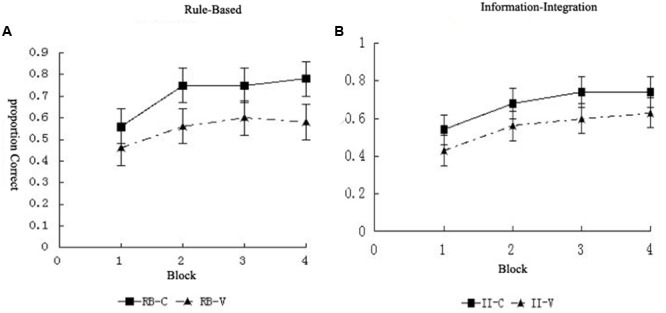
**The categorization accuracy of the (A)** RB and **(B)** II category structures in the visuospatial working memory condition.

We conducted a 2 (category structure) × 2 (condition) × 4 (block) mixed design analysis of variance. This revealed a main effect of block, *F*(3,258) = 74.76, *p* < 0.001, $\eta_{p}^{2}$ = 0.47, indicating learning, and a main effect of condition, *F*(1,86) = 14.52, *p <* 0.001, $\eta_{p}^{2}$ = 0.14, indicating superior accuracy overall for the control condition compared to the visuospatial working memory condition. There was no main effect of category structure, *F <* 1, and no significant interactions between block and category structure, *F <* 1, between block and condition, *F*(3,258) = 1.44, *p =* 0.232, between category structure and condition, *F <* 1, or between block, category structure, and condition, *F*(3,258) = 1.13, *p =* 0.336 (**Figure 7**).

Furthermore, for the RB category structure, we performed a 2 (condition) × 4 (block) repeated-measures analysis of variance (**Table 4**). The results showed that the main effect of the block was significant, *F*(3,123) = 25.36, *p* < 0.001, $\eta_{p}^{2}$ = 0.38, indicating the existence of a learning effect. The main effect of the condition was also significant, *F*(1,41) = 7.87, *p* = 0.008, $\eta_{p}^{2}$ = 0.16; the categorization results of participants in the RB-V group were significantly lower than those of participants in the RB-C group. In addition, the interaction between the condition and block was not significant, *F*(3,123) = 1.76, *p* = 0.158. The results showed that the visuospatial working memory task impaired the learning performance in the RB category structure.

**Table 4:** Effects of working memory on the II and RB category structures under the condition of simultaneous presentation (*M ± SD*).

	1	2	3	4
RB-C	0.56 ± 0.16	0.75 ± 0.17	0.75 ± 0.17	0.78 ± 0.19
RB-V	0.46 ± 0.16	0.56 ± 0.21	0.60 ± 0.25	0.58 ± 0.27
II-C	0.54 ± 0.12	0.68 ± 0.16	0.74 ± 0.16	0.74 ± 0.15
II-V	0.43 ± 0.14	0.56 ± 0.19	0.60 ± 0.22	0.63 ± 0.22

*RB and II represent the rule-based category structure and the information-integration category structure, respectively. RB-C represents the rule-based category structure control group, RB-V represents the rule-based category structure experimental group, II-C represents the information-integration category structure control group, and II-V represents the information-integration category structure experimental group. The numbers 1, 2, 3, and 4 represent the four blocks of the learning process.*

For the II category structure, we also performed a 2 (condition) × 4 (block) repeated-measures analysis of variance. The results showed that the main effect of the block was significant, indicating the existence of a learning effect, *F*(3,135) = 58.41, *p* < 0.001, $\eta_{p}^{2}$ = 0.57. In addition, the main effect of the condition was significant, *F*(1,45) = 6.49, *p* = 0.014, $\eta_{p}^{2}$= 0.13, indicating that the results of participants in the II-C group were significantly higher than those of participants in the II-V group. These findings suggest that the visuospatial working memory task can similarly affect the learning of the II category structure. The interaction between the condition and block was not significant, *F*(3,135) = 0.31, *p* = 0.817.

In Experiment 3, the dual-task paradigm with simultaneous presentation was employed, in which visuospatial working memory was integrated into the category learning task. The results revealed that visuospatial working memory interferes with both RB and II category learning, which means that any visual working memory task that involves visual resources, such as the one used in Experiment 2, also disrupts the II category learning system. This finding help to clarify the workings of the implicit system. This system could certainly be a procedural system but it could also rely heavily on visual resources to learn how to classify visually similar stimuli into the same category.

## General Discussion

Previous research has made clear the importance of working memory for RB categories (Zeithamova and Maddox, 2006, 2007; DeCaro et al., 2008; Minda et al., 2008; Rabi et al., 2015). We were interested in further exploring the effect of visuospatial working memory on RB and II category learning, especially investigating the role of visual processing and executive functioning. In Experiment 1, the dual-task paradigm with sequential presentation was adopted to investigate the influence of visuospatial working memory on implicit and explicit category learning. The results showed that visuospatial working memory interferes with RB but not II category learning. In Experiment 2, the dual-task paradigm with simultaneous presentation was used, in which the categorization task was integrated into the working memory task. The results indicated that visuospatial working memory affects II category learning but not the RB learning system. In Experiment 3, the dual-task paradigm with simultaneous presentation was employed, in which visuospatial working memory was integrated into the category learning task. The results revealed that visuospatial working memory interferes with both RB and II category learning. Through these three experiments, we found that, regarding the RB category structure, executive function is the main mechanism by which visuospatial working memory influences the rules and the discovery of the rules but not the category representation. In addition, regarding the II category structure, visual processing mainly operates on the category representation, which interferes with the connection between the interference space and the specific action.

### Visuospatial Working Memory Affects the RB Category Learning

Our study showed that visuospatial working memory affects the RB category learning, and that working memory plays an important role during this process. During the process of the RB category learning, working memory is used to update and retrieve the rules from memory that are tested by feedback, while executive function is also needed to restrain the interference of irrelevant dimensions. Presenting the visuospatial working memory tasks sequentially occupies working memory and, as a result, the verification of rules conducted by the feedback is interfered with (Zeithamova and Maddox, 2007; Grimm and Maddox, 2013). When the visuospatial working memory task was embedded in the category learning (as in Experiment 3), we believe that the visuospatial working memory task mainly interfered in the discovery of the categorization rules; as soon as the participants were successful in identifying the categorization rules, they were able to learn successfully and their accuracy increased significantly.

However, when the category learning task was embedded in the visuospatial working memory task, the results showed that the effect of visuospatial working memory on RB category learning disappeared. That is, visuospatial working memory did not affect the RB category structure. It is worth noting that, although they are both task paradigms with simultaneous presentation, the existing studies suggest that, compared with the condition in which the visuospatial working memory task is integrated into the category learning task (as in Experiment 3), the condition in which the category learning task is integrated into the visuospatial working memory task requires a higher level of executive function. This is because, during the process of accomplishing the category learning task, participants need to use working memory consistently to retain the beginning of the visuospatial working memory task (Miles and Minda, 2011).

Therefore, according to the assumption of the COVIS model, the RB category learning should be hindered more heavily when the category learning task is integrated into the visuospatial working memory task than when the visuospatial working memory task is integrated into the category learning task. However, our experimental results contradicted this. Why was there such a result? We think that this was caused by the fact that working memory or executive function can affect a specific phase of cognitive processing during category learning. Although the condition in which the category learning task is integrated into the visuospatial working memory task requires more executive function, in the RB category structure learning, the perception of the category stimuli does not rely on executive function. Zeithamova and Maddox (2007) indicated that visuospatial working memory is more likely to be used to represent the optimal categorization criteria, while during explicit category learning, it uses assumptions to examine the categorization rules and relies on working memory to keep these categorization rules in mind.

### Visuospatial Working Memory Affects the II Category Learning

According to the COVIS model, working memory does not affect the learning of the II category structure, because II category learning establishes a connection between a specific perceptual area of the brain and a specific action, relying on the implicit category learning system. However, our results showed that the visuospatial working memory task also affected the RB and II category structure. When simultaneous tasks were used, the executive function of the visuospatial working memory task, no matter whether at a high or low level, affected the results of the II category structure, which indicates that executive function is not the key factor that affects the II category structure, whereas the visual processing of the visuospatial working memory task plays an important role. Dunn et al. (2012) found that the type of grating mask presented after the category stimuli affected the perceptual representation of the implicit category learning, which to some extent indicates that in the learning of the II category structure, visual processing is more likely to affect the original perceptual representation of the category stimuli. In addition, it has been found that the visuospatial working memory of children is slower than that of adults, but the visual processing capacity of children is fully developed and is not lower than that of adults (Huang-Pollock et al., 2011). Minda et al. (2008) showed that the learning results of children (5–7 years old) for the II category structure was not significantly different from that of adults.

How does visual processing affect the implicit category system? We think that the visual processing of the visuospatial working memory task affects the different processing stages of the category learning. By comparing Experiment 1 with Experiment 3, we can observe that, in the condition of the sequential presentation of the dual tasks, the visuospatial working memory task did not affect the II category structure because the II category learning depends on the connection between a specific area of the brain and a specific action.

The primary role of feedback is to provide instant reinforcement, and this stage of forming the category criterion does not necessarily rely on working memory and visual processing; it is more likely to involve implicit unconscious processing. As a result, the visuospatial working memory task with sequential presentation does not influence the II category learning, whereas when dual tasks with simultaneous presentation are used, the intensity of the executive function when the visuospatial working memory task is integrated into the category learning task is the same as when the tasks are presented sequentially. This indicates that when visuospatial working memory influences the II category structure, it is the location of the visuospatial working memory rather than the intensity of the executive function that is actually operating.

By comparing Experiment 2 with Experiment 3, we can observe that the II-V group is always better than the II-C group, which indicates that presenting the visuospatial working memory task after the II category structure has a negative influence on the category learning results from the very beginning of the learning. This suggests that an individual is more likely to be influenced by visual processing during the stage of category representation. However, when the category learning was integrated into the visuospatial working memory task, as for the overall learning cycle, there was no significant difference in results between the II-C and II-V groups in Block 1, and the results of the II-V group were significantly higher than those of the control group from Block 2 onward. Visuospatial working memory involves visual processing and visual perception, while the implicit category system needs to project a specific representation to a specific area of the brain and depends on the visual and perceptual memory systems to improve the stimulus representation that has been recognized and processed, especially to distinguish between representations that are similar but not the same. Therefore, when the visuospatial working memory task is presented at the very beginning, it does not affect the process of the representation of stimuli from different category learning phases, but it does affect the establishment of the connection between the perceptual space and the specific action (i.e., it affects the representation of the category criterion).

## Conclusion

(1) Visuospatial working memory affects RB and II category learning.
(2) Regarding the RB category structure, visuospatial working memory influences the discovery of rules in particular.
(3) Regarding the II category structure, visual processing primarily operates on the category representation, which interferes with the connection between the perceptual space and the specific action.

## Ethics statement

The study was approved by the ethical committee of Guangzhou University. Because the data were analyzed anonymously, and no apparent ethical research complication with participation could be identified, informed oral consent was recommended and obtained from participants before data collection. Participants were given the opportunity to refuse to participate, to omit questions or to withdraw from the study at any time without penalization.

## Author Contributions

QX and HS conceived and designed experiments. HS carried out experiments and analyzed experimental results. HS and QX wrote the manuscript.

## Conflict of Interest Statement

The authors declare that the research was conducted in the absence of any commercial or financial relationships that could be construed as a potential conflict of interest.

## Abbreviations

COVIS: competition between verbal and implicit systems model
II: information-integration
II-C: information-integration category structure control group
II-V: information-integration category structure experimental group
RB: rule-based
RB-C: rule-based category structure control group
RB-V: rule-based category structure experimental group.

## References

[B1] Ashby F. G. Alfonso-Reese L. A. Turken A. U. Waldron E. M. (1998). A neuropsychological theory of multiple systems in category learning. *Psychol. Rev.* 105 442–481. 10.1037/0033-295X.105.3.442 9697427

[B2] Baddeley A. D. Logie R. H. (1999). “Working memory: the multiple-component model,” in *Models of Working Memory: Mechanisms of Active Maintenance and Executive Control* eds Miyake A. Shah P. (New York, NY: Cambridge University Press) 28–61.

[B3] Brainard D. H. (1997). The psychophysics toolbox. *Spat. Vis.* 10 433–436. 10.1163/156856897X00357 9176952

[B4] Casale M. B. Ashby F. G. (2008). A role for the perceptual representation memory system in category learning. *Percept. Psychophys.* 70 983–999. 10.3758/PP.70.6.983 18717385 PMC2562695

[B5] DeCaro M. Thomas R. Beilock S. (2008). Individual differences in category learning: sometimes less working memory capacity is better than more. *Cognition* 107 284–294. 10.1016/j.cognition.2007.07.001 17707363

[B6] Dunn J. C. Newell B. R. Kalish M. (2012). The effect of feedback delay and feedback type on perceptual category learning: the limits of multiple systems. *J. Exp. Psychol. Learn. Mem. Cogn.* 38 840–850. 10.1037/a0027867 22746952

[B7] Filoteo J. V. Lauritzen S. Maddox W. T. (2010). Removing the frontal lobes: the effects of engaging executive functions on perceptual category learning. *Psychol. Sci.* 21 415–423. 10.1177/0956797610362646 20424079 PMC2861791

[B8] Goldman-Rakic P. S. (1998). “The prefrontal landscape: implications of functional architecture for understanding human mentation and the central executive,” in *Prefrontal Cortex: Executive and Cognitive Functions* eds Roberts A. C. Robbins T. W. Weiskranz L. (New York, NY: Oxford University Press) 87–102. 10.1098/rstb.1996.0129 8941956

[B9] Grimm L. R. Maddox W. T. (2013). Differential impact of relevant and irrelevant dimension primes on rule-based and information-integration category learning. *Acta Psychol.* 144 530–537. 10.1016/j.actpsy.2013.09.005 PMC3874386 24140820

[B10] Huang-Pollock C. L. Maddox W. T. Karalunas S. L. (2011). Development of implicit and explicit category learning. *J. Exp. Child Psychol.* 109 321–335. 10.1016/j.jecp.2011.02.002 21377688 PMC3069659

[B11] Maddox W. T. Ashby F. G. Ing A. D. Pickering A. D. (2004). Disrupting feedback processing interferes with rule-based but not information-integration category learning. *Mem. Cogn.* 32 582–591. 10.3758/BF03195849 15478752

[B12] Miles S. J. Matsuki K. Minda J. P. (2014). Continuous executive function disruption interferes with application of an information integration categorization strategy. *Atten. Percept. Psychophys.* 76 1318–1334. 10.3758/s13414-014-0657-8 24719236

[B13] Miles S. J. Minda J. P. (2011). The effects of concurrent verbal and visual tasks on category learning. *J. Exp. Psychol. Learn. Mem. Cogn.* 37 588–607. 10.1037/a0022309 21319921

[B14] Minda J. P. Desroches A. S. Church B. A. (2008). Learning rule-described and non-rule-described categories: a comparison of children and adults. *J. Exp. Psychol. Learn. Mem. Cogn.* 34 1518–1524. 10.1037/a0013355 18980411

[B15] Patalano A. L. Smith E. E. Jonides J. Koeppe R. A. (2001). PET evidence for multiple strategies of categorization. *Cogn. Affect. Behav. Neurosci.* 1 360–370. 10.3758/CABN.1.4.360 12467087

[B16] Rabi R. Miles S. J. Minda J. P. (2015). Learning categories via rules and similarity: comparing adults and children. *J. Exp. Child Psychol.* 131 149–169. 10.1016/j.jecp.2014.10.007 25558860

[B17] Rabi R. Minda J. P. (2014). Rule-based category learning in children: the role of age and executive functioning. *PLoS ONE* 9:e85316 10.1371/journal.pone.0085316 PMC3906381 24489658

[B18] Richler J. J. Palmeri T. J. (2014). Visual category learning. *Wiley Interdiscip. Rev. Cogn. Sci.* 5 75–94. 10.1002/wcs.1268 26304297

[B19] Stanton R. D. Nosofsky R. M. (2007). Feedback interference and dissociations of classification: evidence against the multiple-learning-systems hypothesis. *Mem. Cogn.* 35 1747–1758. 10.3758/BF03193507 18062551

[B20] Sun H. L. Xing Q. (2014). The influences of feedback on perceptual category learning and its’cognitive and neurophysiological mechanisms. *Adv. Psychol. Sci.* 22 67–74. 10.3724/SP.J.1042.2014.00067

[B21] Worthy D. A. Markman A. B. Maddox W. T. (2013). Feedback and stimulus-offset timing effects in perceptual category learning. *Brain Cogn.* 81 283–293. 10.1016/j.bandc.2012.11.006 23313835 PMC3560315

[B22] Xing Q. Sun H. L. (2015). The effect of feedback delay and mask type on perceptual category learning. *J. Psychol. Sci.* 38 521–528.

[B23] Yamauchi T. Markman A. B. (1998). Category learning by inference and classification. *J. Mem. Lang.* 39 124–148. 10.1006/jmla.1998.2566

[B24] Zaki S. R. Kleinschmidt D. F. (2014). Procedural memory effects in categorization: evidence for multiple systems or task complexity? *Mem. Cogn.* 42 508–524. 10.3758/s13421-013-0375-9 24217892

[B25] Zeithamova D. Maddox W. T. (2006). Dual-task interference in perceptual category. *Learn. Mem. Cogn.* 34 387–398. 10.3758/BF03193416 16752602

[B26] Zeithamova D. Maddox W. T. (2007). The role of visuo-spatial and verbal working memory in perceptual category learning. *Mem. Cogn.* 35 1380–1398. 10.3758/BF03193609 18035635

